# Determination of resilience of a panel of broadly neutralizing mAbs to emerging variants of SARS-CoV-2 generated using reverse genetics

**DOI:** 10.1016/j.isci.2025.112451

**Published:** 2025-04-16

**Authors:** Madeeha Afzal, Diana Melnyk, Thomas Courty, Lisa Schimanski, Michelle Hill, Stuart Neil, Tiong Kit Tan, William S. James

**Affiliations:** 1 James & Lillian Martin Centre, Sir William Dunn School of Pathology, University of Oxford, South Parks Road, OX1 3RE Oxford, UK; 2 MRC Translational Immune Discovery Unit, MRC Weatherall Institute of Molecular Medicine, John Radcliffe Hospital, University of Oxford, OX3 9DS Oxford, UK; 3 Department of Infectious Diseases, School of Immunology & Microbial Sciences, King’s College London, SE1 9RT London, UK; 4 Chinese Academy of Medical Sciences Oxford Institute, Nuffield Department of Medicine, University of Oxford, OX3 7BN Oxford, UK

**Keywords:** Biological sciences, Genetics, Immunology, Immune system evolution, Immunity

## Abstract

SARS-CoV-2 continues to evolve, and its emerging variants might escape the immune responses generated by existing vaccines and therapeutic mAbs. Accordingly, rapid analysis of their possible neutralization phenotype is essential and can be facilitated by reverse genetics (RGs) systems to regenerate viruses with variant-specific substitutions. Here, we efficiently generate a panel of recent variants of SARS-CoV-2 (Omicron XBB.1.16, EG.5.1, BA.2.86, and JN.1) using a substantially optimized circular polymerase extension reaction (CPER) RGs system. Neutralization potency was analyzed for mAbs targeting different regions of spike protein. mAbs P4-J15, C68.61, S2X259, and IY-2A IgG were able to neutralize all recent viruses. However, S309, which was previously used to treat infection and targets the outer face of RBD, showed ∼75-fold reduction in potency versus JN.1. Moreover, C68.59, which targets the SD1 region of the CTD, was unable to neutralize either BA.2.86 or JN1, which share the E554K substitution in SD1. CPER RGs system and microneutralization assays can be adopted as effective tools to evaluate the efficacy of therapeutic mAbs against emerging variants in a time-responsive manner.

## Highlights

• CPER reverse genetics system expedites mAb evaluation against emerging SARS-CoV-2 variants
• S309 mAb shows a significant reduction in potency against SARS-CoV-2 Omicron JN.1
• C68.59 mAb did not retain potency against SARS-CoV-2 Omicron BA2.86 and JN.1
• P4-J15, IY-2A, S2X259, and C68.61 mAbs can neutralize recent SARS-CoV-2 variants

## Graphical abstract

**Figure:**
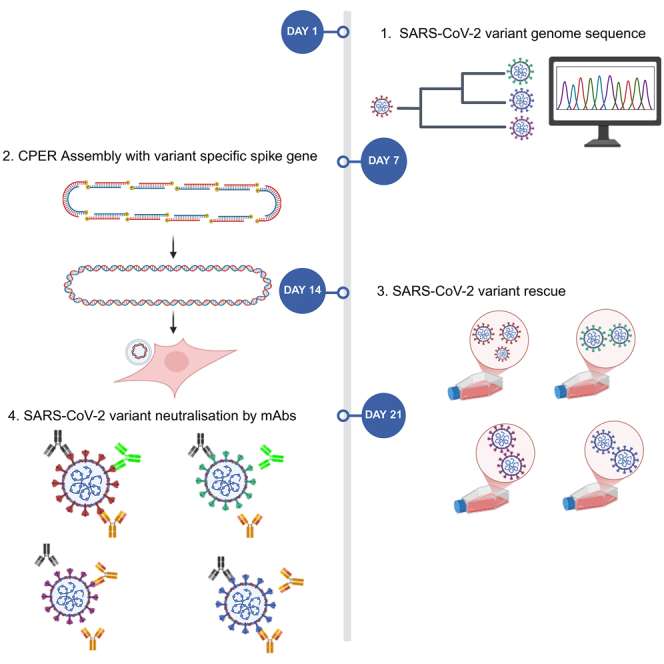

## Introduction

As the severe acute respiratory syndrome coronavirus 2 (SARS-CoV-2) continues to evolve, the countermeasures such as the vaccines, antiviral drugs, and the diagnostic assays also need to be re-evaluated and often updated against emerging variants. Emerging variants of SARS-CoV-2 often have mutations in its B-cell epitopes that may alter the binding affinity of neutralizing antibodies and often result in immune evasion. This phenomenon can significantly impact the effectiveness of immune responses, whether from natural infection or vaccination, as the immune system may no longer recognize and effectively neutralize the variant virus. The host immune system also exerts a selective pressure on the virus, and hence the variants that can escape immune detection have a survival advantage and are more likely to propagate. Vaccination campaigns and natural infections also increase this pressure, leading to the emergence of variants with modified epitopes. Such variations in epitopes can have implications on vaccine efficacy and can impact the sensitivity of the diagnostic assays as well. In particular, vaccines designed to target variable epitopes may become less effective against emerging variants if those epitopes undergo significant changes. This rapid evolution has made it difficult to study responses to the most relevant variants in real time. This necessitates development of tools for quick analysis of emerging variants.

The reverse genetics (RG) systems can be used to rapidly engineer viruses with desired mutations to study the effect of these mutations without having to generate the wild type viruses from clinical isolates. Several methods of RG engineering of SARS-CoV-2 have been described including *in vitro* ligation and transcription,^1^^,^^2^ bacterial artificial chromosome (BAC)^3^^,^^4^ based methods, infectious sub-genomic amplicon based method,^5^^,^^6^ circular polymerase extension reaction (CPER) mediated assembly,^7^ vaccinia virus–mediated recombination,^8^ yeast artificial chromosome (YAC) based transformation associated recombination (TAR),^9^ etc. CPER and transfection of the resulting circular genome into susceptible producer cells is an appealing method as it is quick and bacteria-free. Several modifications of this method have also been reported including the nick ligation of CPER reaction^10^ and the use of co-culture.^11^ In Torii et al.’s method, CPER products were transfected into HEK293-3P6C33 cells which are tetracycline inducible ACE2 and TMPRSS2 expressing, IFNAR1-deficient HEK293 cells. Amarilla et al. tried a coculture of CPER-transfected HEK293T cells with Vero E6. In this study, we use a modified HEK293T17 cell line, which constitutively expresses SV40 large tumor antigen (LTag),^12^ with IFNAR1 knock out, and ACE2 and TMPRSS2 knocked in for initial transfection, followed by a co-culture on Vero E6-TMPRSS2 cells after 48 h of transfection. In Torii et al.’s method, the linker comprised of 3′ 43 nt of SARS-CoV-2, BGH polyA signal, HDVr, CMV promoter and the 5′ 25 nt of SARS-CoV-2. We have used a modified linker comprising SV40 origin of replication and an SV40 poly A signal in addition to the modified HDVr sequence, CMV Promotor and 3′ and 5′ UTR sequences of SARS-CoV-2. The rationale for introduction of SV40 ori in the linker region is to improve the replication of the CPER construct in the transfected cells by the help of the constitutively expressed LTag.^12^^,^^13^ We have also included the SV40 intron in the linker as it is thought to enhance expression.^14^ We have analyzed the efficiency of both the nick ligated and unsealed CPER methods together with a modified co-culture method and a modified linker fragment, and were able to rescue the virus with high titer as early as the 3rd day post co-culture. We have also used this method to successfully develop a quick workflow to generate the chimeric SARS-CoV-2 viruses bearing spike proteins of newly emerging variants.

Using these RG viruses, we validated and evaluated the neutralization activity of recent SARS-CoV-2 variants against several potent neutralising mAbs targeting epitopes of spike protein. The S1 subunit of the spike protein has two important domains that are targets of monoclonal antibodies (mAbs), namely the NTD and RBD. Antibodies binding the spike RBD were initially^15^ grouped into four classes on the basis of their mode of binding to the S protein. Recently several other neutralizing antibodies have been identified that do not fall into these four classes.^16^^,^^17^The NTD-targeting antibodies^18^^,^^19^^,^^20^^,^^21^ often bind the NTD supersite. Moreover, three main classes of S2 binding antibodies have been described in the literature: those binding the fusion peptide and adjacent S2' cleavage site^22^^,^^23^ and those binding the S2 stem proximal to the viral membrane^24^^,^^25^^,^^26^^,^^27^ and a third class of S2 antibody that binds the highly conserved S2 hinge region, which converts from a bent hairpin to extended alpha helix during the pre-to-post-fusion spike conformational change.^28^ Here, a panel of mAbs targeting various regions of spike protein was used to study the neutralization profile. These included IY-2A (RBD Class 4 mAb),^29^ P4J15 (ACE-2 mimetic mAb),^30^ C68.59 (SD1 binding), and C68.61 (RBD Class 3 based on epitope similarity with S309)^31^ but also referred to as Class 5 like recently,^32^ S2X259 (RBD Class 1/4 mAb),^33^ and S309 (RBD class 3)^34^ and found that, for the most part, variation had limited effect on neutralization of these mAbs compared to the original WH-1 variant, although BA.2.86 and JN.1 evaded neutralization by C68.59.

The optimized CPER protocol incorporates a modified linker fragment bearing SV40 3′ Splice site, SV40 poly(A) 1, SV40 origin of replication, and CMV promoter. It also utilizes 5′phosphorylated DNA fragments generated by using 5′phosphorylated primers in the PCR, followed by nick-ligation, and the use of modified HEK- HEK293T/17 (TMPRSS2+, ACE2+, IFNAR1 KO) cell line for original transfection, followed by a co-culture with Vero E6 TMPRSS2 cells, for generation of genetically engineered SARS-CoV-2. The workflow for generating chimeric viruses bearing spikes of emerging variants on the USA-WA1 backbone can facilitate the rapid analysis of *in vitro* neutralization potency of the mAbs against emerging variants of SARS-CoV-2.

## Results

### Optimization of CPER RG method

Our investigations to improve the efficiency of the method were aimed at.

#### Comparison of nick-ligated and unsealed CPER reactions conducted with 5′ phosphorylated and unphosphorylated DNA fragments, respectively

**Figure 1:**
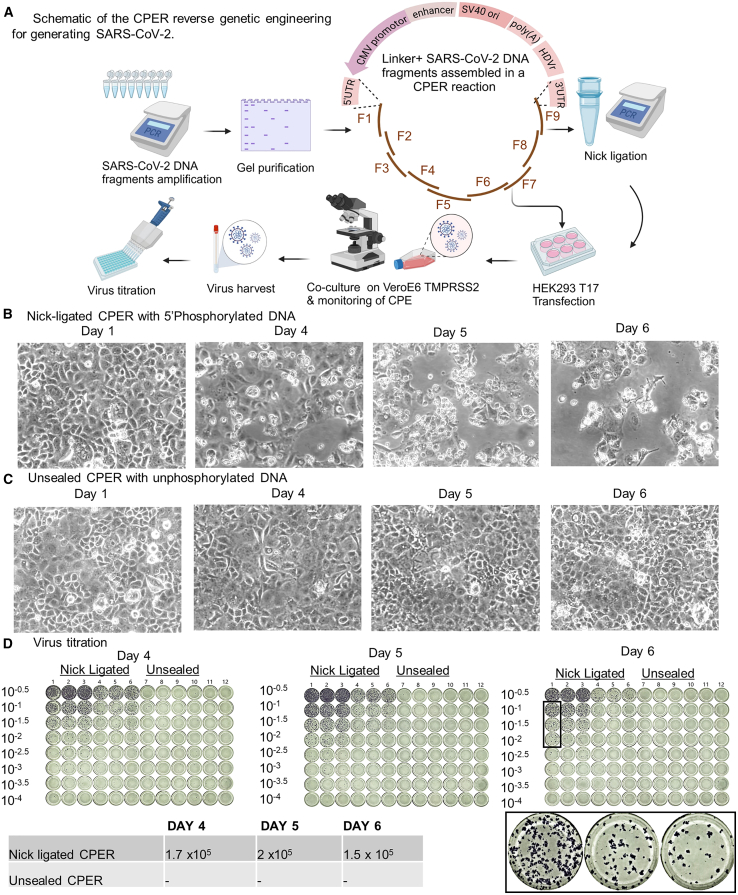
Comparison of nick-ligated and unsealed CPER reactions conducted with 5′phosphorylated and unphosphorylated DNA fragments respectively (A) An overview of the CPER reverse genetic engineering protocol for generating SARS-CoV-2. (B) Nick-ligated CPER shows cytopathic effects (CPE) as early as day 4 post co-culture. (C) Unsealed CPER using unphosphorylated DNA fragments. CPE were not observed. (D) Titration of the harvested cell-culture supernatant with FFU assay shows that virus was produced only in case of nick-ligated CPER reaction.

Photomicrographs of the VeroE6 TMPRSS2 cells co-cultured with the transfected HEK293T/17 cells for demonstration of cytopathic effects (CPE) are shown in Figure 1. CPER based RG engineered SARS-CoV-2 USA-WA1 rescue is evident by CPE. Culture supernatant samples harvested on the 4th, 5th, and 6th days post-co-culture were titrated with the FFU assay, and SARS-CoV-2 virus was recovered on all the three days in case of nick-ligated CPER reaction, but not in case of unsealed CPER reaction.

#### Comparison of nick-ligated and unsealed CPER reactions carried out with 5′phosphorylated DNA fragments in both cases

**Figure 2:**
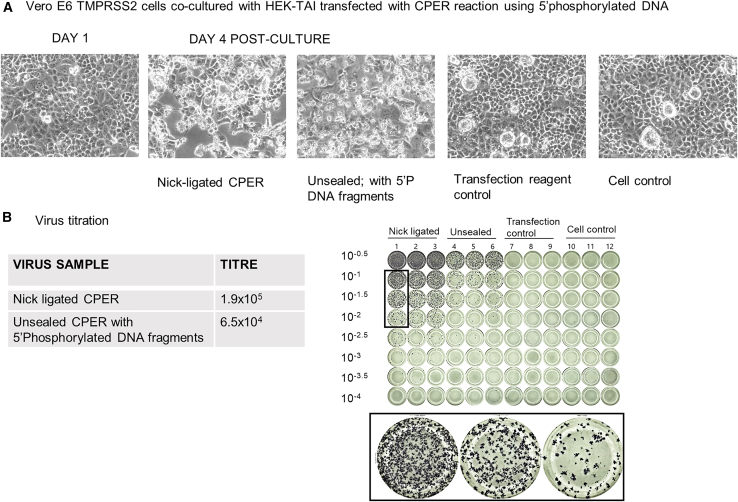
Comparison of nick-ligated and unsealed CPER reactions carried out with 5’phosphorylated DNA fragments in both cases (A) Nick-ligated CPER and unsealed CPER using 5′phosphorylated DNA fragments. CPE can be seen in both the cases. (B) Titration of the harvested cell-culture supernatant with FFU assay shows that virus was produced in both the cases of nick-ligated and unsealed CPER reactions.

When 5′phosphorylated fragments were used to set up the CPER reaction, SARS-CoV-2 virus was recovered in case of unsealed CPER reaction as well, as can be seen by the CPE in the photomicrographs shown in Figure 2. However, virus titration by FFU assay revealed that the yield of the virus from the nick-ligated reaction was higher as compared to that from the unsealed CPER.

#### Comparison of direct transfection of VeroE6 TMPRSS2 cells versus the co-culture of VeroE6 TMPRSS2 cells with the transfected HEK293T17 cells

When the Vero E6 TMPRSS2 cells were transfected with the CPER products, we were able to rescue SARS-CoV-2 virus on day 6 post transfection with a titer of 1 × 10^6^ FFU/mL. We can conclude that the modification of linker by including SV40 ori did not have any negative impact on virus rescue upon direct transfection in VeroE6 TMPRSS2 cells. However, as the transfection was typically done in 6 well plate, the volume of the harvested virus is only 1.5mL. In comparison, when we performed the co-culture experiment in T75 flasks, we were able to generate 10mL of harvested virus with a titer of 1 x 10^6^ FFU/mL on day 6 post co-culture (day 8 post-transfection). These data are shown as Figure S4.

### Preparation of spike chimeric viruses using CPER RG method

**Figure 3:**
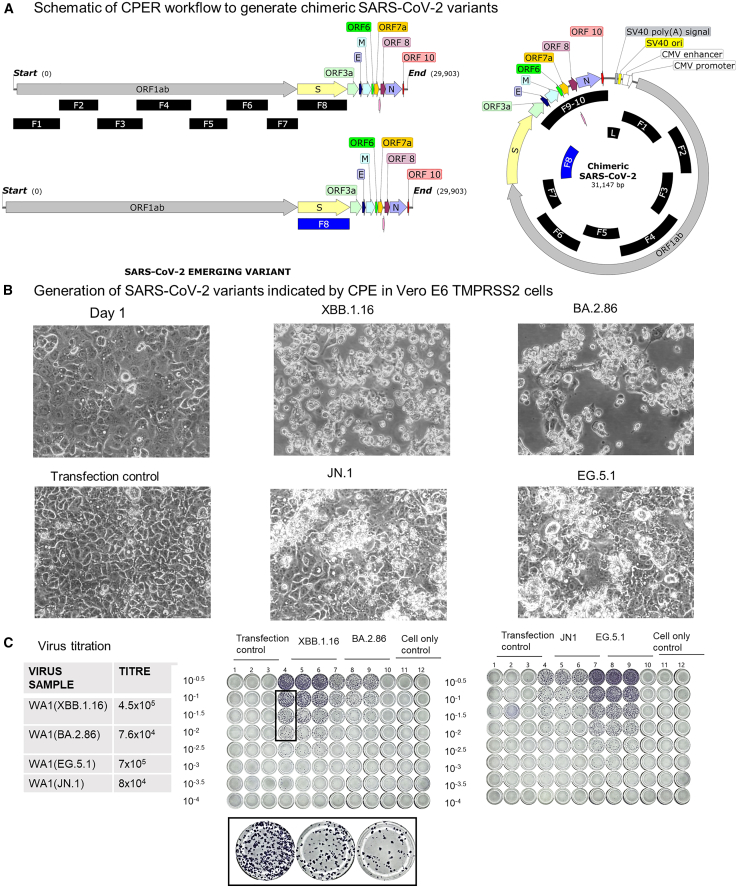
Generation of chimeric SARS-CoV-2 virus using CPER reverse genetics system (A) CPER workflow to generate chimeric SARS-CoV-2. Schematic of CPER assembly using fragment 8 from emerging variants and fragments 1–7 and 9–10 from USA-WA1 with the linker fragment to generate chimeric virus presenting spikes of a recent variant. See also Figure S1. (B) Microscopy images of Vero-E6 TMPRSS2 cultures with visible cytopathic effects indicating generation of chimeric virus bearing spike of recent variant. (C) Titration of the harvested cell culture supernatant with FFU assay confirmed generation of high titer virus.

The CPER RG engineered SARS-CoV-2 viruses were rescued from the cell culture supernatant when CPE were visible in the Vero E6 TMPRSS2 cell cultures as shown in Figure 3B. The harvested samples were titrated with the FFU assay as shown in Figure 3C.

### Virus neutralization assays

**Figure 4:**
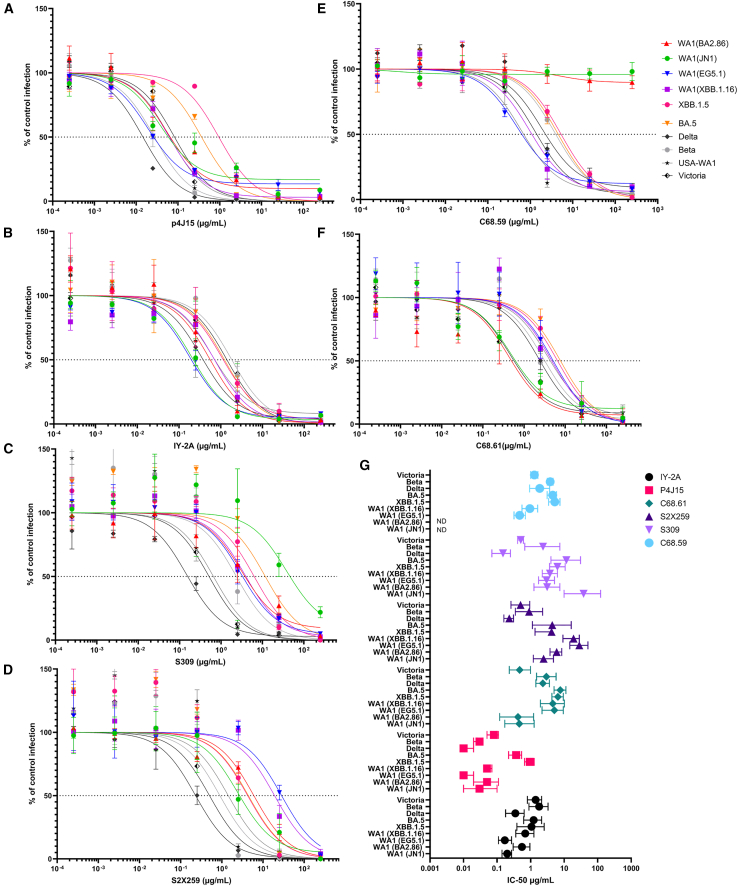
Virus neutralization assays. The neutralization profile of mAbs against SARS-CoV-2 variants (A) The neutralization profile of P4J15 against SARS-CoV-2 variants. (B) The neutralization profile of IY-2A against SARS-CoV-2 variants. (C) The neutralization profile of S309 against SARS-CoV-2 variants. (D) The neutralization profile of S2X259 against SARS-CoV-2 variants. (E) The neutralization profile of C68.59 against SARS-CoV-2 variants. (F) The neutralization profile of C68.61 against SARS-CoV-2 variants. (G) Best fit IC50 values and the confidence limits for the neutralizing mAbs.

Virus neutralization assays were performed to determine the neutralization potency of each mAb against each variant. Spot counts in the presence of known concentration of mAb were compared with the spot counts in the wells without any antibody to generate IC50 values for the mAbs against each variant. Figure 4. The assays were done in three replicates and at each dilution of mAb, the mean value in comparison with the control is shown, with the errir bars indicating standard deviation.

C68.59, which binds to a rare epitope in the SD1 region downstream of RBD has high neutralization potency and breadth across SARS-CoV-2 VOCs as reported previously.^31^ Our results indicate that C68.59 did not retain potency against recent, dominant variants BA2.86 and JN.1.

C68.61 has retained potency against all the previous SARS-CoV-2 variants, and it shows improved neutralization of the recent variants BA.2.86 and JN.1. C68.61 was notably 10-fold more potent against these more recent VOCs than against XBB.1.16 and EG.5.1.

S309, being the active ingredient in sotrovimab, has shown some weak neutralization against JN.1 with 10-fold reduction compared to XBB.1.16 and 75-fold reduction as compared to the Victoria. S2X259 has neutralized all the emerging variants with varying IC50. However, the neutralization potency for the recent variants JN.1 and BA.2.86 is improved as compared to EG5.1 and XBB.1.16. In our assay, P4J15 and IY-2A have retained neutralization potency against all the emerging variants. IY-2A was found to be 2-fold more potent against BA.2.86 and 5-fold more potent against JN.1 as compared to XBB.1.5. Similarly, P4J15 was found to be 18-fold more potent against BA.2.86 and 31-fold more potent against JN.1 as compared to XBB.1.5.

To quantify the overall potency of the mAbs against SARS-CoV-2, we calculated the geometric mean of the IC50s (GM) across all SARS-CoV-2 viruses tested. P4J15 had the highest overall potency [GM = 0.06 μg/mL], followed by IY-2A [GM = 0.63 μg/mL] and C68.61 [GM = 2.08 μg/mL]. For C68.59, we set the IC50 for those viruses with no neutralization activity to the highest mAb concentration tested. Due to its high potency against the other VOCs, the GM was 5.68 μg/ml; however, its lack of breadth against the recently circulating Omicron JN.1 and BA.2.86 variants makes it a poor candidate for therapeutic use currently. S2X259 [GM = 2.91 μg/mL] performed well against all the variants. S309 [GM = 2.96 μg/mL] seems to have a reduced potency against JN.1 variant.

Design of a mAb cocktail should also carefully consider any competition among these mAbs. It has been previously studied^31^ that C68.61 competed with S309 due to a partially overlapping epitope within the RBD core region.

**Figure 5:**
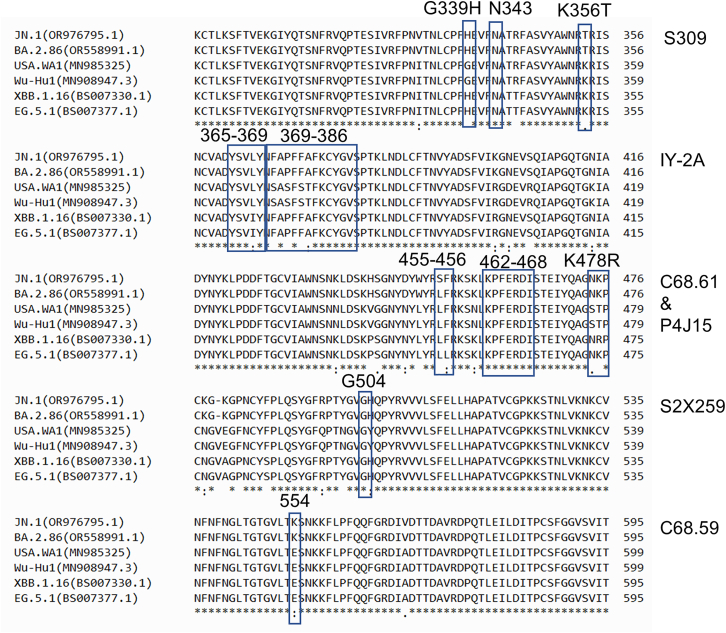
Multiple sequence alignment of the spike protein from SARS-CoV-2 variants Epitopes and escape mutations for the mAbs are indicated. See also Figures S2 and S3 for structural illustration of epitopes.

We also performed the spike sequence comparison to identify possible mutations that explain the genetic basis for any loss of neutralization potency of the mAbs Figure 5.

Escape mutants for C68.59 have been previously^31^ mapped to five key residues (in WH-1 numbering) i.e., E554, K558, R577, E583, and L585. We performed the sequence analysis of the recent variants and found that E554K mutation could be responsible for loss of neutralization potency in JN.1 and BA.2.86 as all the other four residues appear to be conserved in the neutralised (XBB.1.5, XBB.1.16, EG.5.1) and non-neutralized variants (JN.1 and BA.2.86).

C68.61 epitope is distant from the ACE2-binding region in the RBD core and is highly conserved across major SARS-CoV-2 VOCs and SARS-CoV-1. Previous studies^31^^,^^32^ have mapped C68.61 escape at sites K462, E465, R466, and I468. These amino acid residues are conserved in all the recent variants which is consistent with the retention of neutralization potency in recent variants.

S309 recognizes an epitope containing a glycan at N343 that is conserved within the Sarbecovirus subgenus, without competing with receptor attachment.^34^ N343 is conserved in all variants including JN1. It has been recently reported^35^ that S309 fails to neutralize BA.2.86 in pseudovirus assay, possibly contributed by a mutation at 339 and 356 position. However, 339 has been mutated in XBB1.16, XBB.1.5, and EG.5.1 as well which are neutralized in the pseudovirus assay and our authentic virus neutralization assay. Position 356 is mutated in both BA2.86 and JN.1. However, in our assay the remarkable drop of IC50 in case of JN.1 as compared to BA.2.86 raises a possibility about the involvement of spike L456S, in the interaction with S309, which is the only residue that differs in between the two variants.

Class 4 mAb IY-2A recognizes a region (the 365–369 helix), which is originally buried but is exposed after a conformational change upon interaction with the mAb.^29^ A linear peptide (residues 369–386) is also involved in the footprint of all class 4 antibodies. It is worth noting that positions 371, 373, and 375 are well known mutation hotspots that compromise neutralizing antibody binding. Sequence analysis indicates that the position 368 has isoleucine in case of XBB.1.5, XBB.1.16, and EG.5.1 and leucine in case of BA.2.86 and JN.1. However, I368L did not cause any loss of neutralisation.

S2X259 recognizes a highly conserved cryptic epitope of RBD, previously termed as antigenic site II, which becomes accessible only when at least two RBDs in the S trimer adopt an open conformation.^36^ Previous studies with deep-mutational scanning and *in vitro* escape selection experiments demonstrate that S2X259 possesses an escape profile that is limited to a single substitution, G504D.^33^ Sequence analysis indicates that G504 is conserved in all the recent omicron variants (BA2, BA4, BA5, XBB1.5, XBB1.16, EG.5.1, BA.2.86, and JN.1).

P4J15 Mab shares 93% of footprint with ACE2.^30^ Sequence analysis highlighted three significant mutations that lie in the footprint i.e., L455S in JN.1, F456L in EG1, and K478R in XBB.1.16. However, these variations had no impact on neutralisation.

## Discussion

During the past few years, extensive progress in the mapping of SARS-CoV-2 spike epitopes has led to the identification of key neutralization epitopes that contribute to protective humoral immune responses. Much of the research for therapeutic mAbs is focused specifically on the RBD of spike protein. The spike protein on the surface of SARS-CoV-2 is essential for productive viral entry into cells and is recognized by the host ACE2 receptor. Serum neutralizing antibodies, produced after natural infection or vaccination, and the therapeutically developed mAbs often bind to the spike protein, preventing its binding to ACE2 or subsequent conformational changes required for infection. However, the virus constantly evolves, acquiring mutations in the spike protein that render it resistant to neutralizing antibodies, and thus potentially making vaccines less effective. Hence, a quick analysis of such escape mutations is crucial for effective control of the pandemic.

Our assay results demonstrate that the mAbs S2X259, IY-2A, C68.61, and P4J15, neutralized all the SARS-CoV-2 variants that we analyzed with similar potency. S309 appears to have reduced neutralization activity against the recent variant JN.1.

The P4J15 epitope overlaps with almost all of the ACE-2 binding region, and although the spike 455 and 456 residues are involved in contact with both, the mutations at these positions did not drive any immune escape for this antibody.

C68.61 binds to a highly conserved epitope in the RBD, hence, it is inherently likely to have a high barrier to viral escape. Another recently described mAb, S2H97, which binds to the same surface of RBD as C68.61, has also demonstrated broad binding across the entire breadth of bat SARS–related coronaviruses and protects hamsters from SARS-CoV-2 challenge.

C68.59 binds to a neutralizing epitope in spike subdomain 1 located in in the CTD of S1 of spike. We have found that recent variants Omicron BA.2.86, and JN.1 evaded neutralization by C68.59. The specific mechanism by which SD1-specific antibodies neutralize SARS-CoV-2 has not been fully elucidated. Another SD1 targeting antibody P008_60^37^ binds an epitope in L3 of SD1 that is not accessible within the canonical prefusion states of the SARS-CoV-2 spike, suggesting a transient conformation of the viral glycoprotein that is vulnerable to neutralization. However, P008_60 and C68.59 have only partial overlap in their epitopes and therefore could have different neutralization mechanisms.

Extensive research efforts have been made to identify mutational hotspots in spike protein that confer escape to neutralizing antibodies.^38^ For example, in addition to the sites discussed in the results section, the E484K mutation which first appeared in the Beta (B.1.351) variants allowed the virus to evade class 2 antibody neutralization. When combined with K417N and N501Y mutations, this immune escape capacity is further enhanced. *In silico* analysis revealed that E484 frequently interacts with antibodies but not with ACE2. Similarly, S494P, reduces antibody neutralization of convalescent and post-immunization sera, particularly when combined with E484K and with mutations able to increase binding to ACE2, such as N501Y. In a similar study^39^ it was found that a mutation E406W remodeled the RBD allosterically, and abrogated binding of two mAbs (REGN10933 and REGN10987). It is interesting to note that E406W is located outside of the epitopes recognized by either of these two mAbs. Other residues, such as L455, F456, F486, and site 487, also impact antibody binding and are very important for studying the FLIP and the recently emerging FLIRT variants. Such synergic mutations provide signatures for immune evasion hotspots, and can be very helpful in guiding the design of antibody cocktails for therapies, vaccines, and diagnostics. The E554K mutation has been found to be responsible for loss of potency against other anti-SD1 mAbs as well,^40^ possibly due to the disruption of salt bridge between E554 and K535 of the spike protein. This mutation is also present in other variants such as FL.10.1, XBB.1.19.1, and JN.4 and hence these variants are very likely to evade the neutralization by C68.59 and other similar SD1 binding mAbs.

Antibody cocktails can be developed for combating the immune escape variants to ensure maximum coverage of mutations responsible for immune escape. However the development of such cocktails of mAbs may also pose additional challenges such as (1) epitope-masking effects that may not allow simultaneous use of two different antibodies binding the same epitope, (2) steric hinderance effects that may not allow simultaneous use of two antibodies with epitopes quite close to each other, (3) unfavourable conformational changes induced by one member of cocktail that may not allow other members to bind effectively and (4) antibody dependant enhancement. For example, from our panel, C68.61 and P4J15 both bind in the RBD core and therefore might compete with each other for binding if both are present in the mAb cocktail, however, this has not been systematically investigated. Also, S309 is known to compete with C68.61^31^ and therefore both of these should not be present together in a mAb cocktail.

Non-neutralizing antibodies can still help in clearing the virus through Fc mediated effector mechanisms like antibody-dependent cellular cytotoxicity (ADCC) and antibody-dependent cellular phagocytosis (ADCP), as documented for S309^41^ and for C68.61.^32^ These processes are not widely studied for the nAbs in our panel especially for the recent variants.

The development of updated vaccines targeting newer variants is an ongoing challenge and would require research into the epitope evolution of SARS-CoV-2. Recent vaccines aim to improve immune responses against the evolving virus. For example, the bivalent vaccines targeting both the ancestral strain and Omicron subvariants have shown better neutralization capacity against new variants compared to older vaccines.

The variant-specific binding and neutralization can be quite helpful for developing variant specific diagnostic assays for clinical and research utilities.

The CPER-based RG system is a quick and efficient tool for SARS-CoV-2 studies. Despite the large size of the viral RNA genome (∼30 kb), infectious full-length cDNA is readily assembled *in vitro* without the need for technically demanding intermediate steps. Overlapping cDNA fragments are generated from viral RNA and assembled together with a linker fragment containing CMV promoter into a circular full-length viral cDNA in a single reaction. We have optimized the method through the use of a modified linker bearing SV40 origin of replication together with the nick-ligation^10^ and the co-culture method,^11^ but we have not yet systematically determined which one(s) of these specific steps is functionally most important for the CPER optimization. It is conceivable that the combination of the new modifications leads to successful virus generation in a shorter time. In current study, modified HEK293T/17 cells were used for initial transfection and were later co-cultured on VeroE6 TMPRSS2 cells. Just like the parent HEK293T cells, HEK293T/17 (ACE2+, TMPRSS2+, IFNAR1 KO) cells not only constitutively express the SV40 large T antigen (LTag) which is required for SV40 DNA replication, but also have the additional modifications i.e.: (1) IFNAR1 knock out (which is helpful in supporting viral infection and preventing the host cell apoptosis), (2) ACE2 knocked in (being a receptor of SARS-CoV-2, presence of ACE2 is very helpful in continued viral infection after transfection), and (3) TMPRSS2 knocked in (is the serine protease that cleaves the viral spike during infection. Presence of TMPRSS2 in the host cell would ensure that the progeny virus has the authentic furin cleavage sequence.) The modified HEK293T/17 cells on their own do not support efficient SARS-CoV-2 rescue upon CPER transfection (data not shown here), however, a co-culture of these on VeroE6 TMPRSS2 cells is helpful in improving the viral titer and the virus can be rescued on 4th day post co-culture. Additionally, Vero E6/TMPRSS2 cells have been used widely for enhanced SARS-CoV-2 isolation,^42^ induce minimal culture adaptation in SARS-CoV-2,^43^ making them a reliable choice for generating SARS-CoV-2 maintaining the virus’s natural characteristics. SARS-CoV-2 isolates that are cultured in the lab may rapidly develop mutations or deletions especially in the Furin cleavage site.^44^^,^^45^^,^^46^ Viral production on TMPRSS2 bearing cells and using a bacteria-free RG system with a quick turn-around time is helpful in minimizing mutations. We have also compared the direct transfection of VeroE6 TMPRSS2 cells with the optimized co-culture method as shown in Figure S4, and the virus was successfully recovered in both cases. Although a direct transfection of VeroE6 TMPRSS2 cells will not benefit from the replication advantage in the HEK293T cells, but it is still advantageous to rescue the virus with authentic furin cleavage site. The co-culture method however can offer the advantage of (1) replication of the CPER product for enhanced copy number and (2) ensuring that the virus does not carry any mutations in the polybasic cleavage site. Our optimized CPER method is a simple method as it uses phosphorylated primers to generate DNA fragments so there is no need to do the kinase treatment afterward. The optimized linker fragment is compatible with both direct transfection in VeroE6 TMPRSS2 cells as well as with the co-culture method using modified HEK293T/17 and VeroE6 TMPRSS2 cells. It is a highly reproducible and efficient method that offers high titer virus in reasonable volume in a quick time. Therefore, our method can be easily adopted by other laboratories, as the high transfection efficiency of modified HEK293T/17 cells, combined with the high permissivity of the co-cultured VeroE6 TMPRSS2 cells to virus replication provides a simple, reliable, and inexpensive method for the large scale production of virus of any desired genotype.

For chimeric virus production, generating mutant spike sequence can be done readily by using commercial synthesis or site-directed mutagenesis, allowing to produce SARS-CoV-2 virus bearing mutations that may be in circulation (such as the variants of concern for SARS-CoV-2), or to evaluate the importance of specific amino acids on receptor binding and entry. The relative ease and short time frame involved in generating single-point mutations allows for the rapid screening of key sites. RG system of SARS-CoV-2 virus can also be very helpful in functional analysis of variants such as changes in growth kinetics, host tissue tropism, changes in response to antiviral drugs,^47^ changes in neutralization potency of mAbs, nanobodies or aptamers; to study function of viral genes, and mechanism of pathogenesis including key events during infection such as interaction with ACE2 receptor and fusogenicity. For instance, Johnson et al.^48^ highlighted the significance of the furin cleavage site in the spike protein for viral infection and pathogenicity. By creating a ΔPRRA mutant virus lacking furin cleavage site, they were able to elucidate its impact on viral behavior. Furthermore, RG systems have been utilized to generate a mouse-adapted strain of SARS-CoV-2, which provided insights into viral replication and pathogenicity in a live animal model.^49^ Moreover, the integration of RG with other methodologies has enhanced the understanding of SARS-CoV-2. For example, Dinnon et al.^50^^,^^51^ combined predictive molecular modeling with RG to alter the RBD of the virus via Q498Y/P499T, facilitating viral infection in mice via mouse ACE2 receptors. This not only helped in understanding viral entry mechanisms but also assisted in the vaccine challenge studies and the evaluation of pegylated interferon lambda-1 as a therapeutic for SARS-CoV-2. RG methods have also been used to generate an avirulent strain for use as a live-attenuated vaccine.^52^^,^^53^

### Limitations of the study

Understanding and monitoring the trends in SARS-CoV-2 evolution of epitopes are essential for developing effective therapeutic mAbs. Due to health and safety restrictions, we are not permitted to isolate any escape mutants grown in the presence of the mAbs, and hence have not studied the mechanistic details of the loss of neutralization for any isolated escape variant. Despite broad neutralization activity, the *in vivo* protective efficacy of the neutralizing antibodies needs to be determined using SARS-CoV-2 virus challenge experiments in animals. Human clinical trials of these therapeutic antibodies can also be helpful in obtaining information about Fc-effector functions like ADCC and ADCP, and can also help us better understand if these mAbs can confer protection *in vivo*, either alone or in combination.

## Resource availibility

### Lead contact

Further information and requests for reagents and resources can be made to the lead contact, Dr. Madeeha Afzal (Madeeha.afzal@path.ox.ac.uk).

### Materials availability

Plasmids and antibodies generated for this study can be made available upon request from the lead contact.

### Data and code availability

This paper does not report original code. IC50 values will be shared by the lead contact upon request. Any other additional data can be provided for reanalysis if requested from the lead contact.

## Acknowledgments

We thank Dr Julie Overbaugh and Dr Jamie Guntheor from Human Biology Division, Fred Hutchinson Cancer Centre, USA, for providing the sequences of C68.59 and C68.61 mAbs and for critical review of the manuscript. We thank Dr Alain R. Townsend from MRC Translational Immune Discovery Unit, MRC Weatherall Institute of Molecular Medicine, John Radcliffe Hospital, University of Oxford and Centre for Translational Immunology, Chinese Academy of Medical Sciences Oxford Institute, University of Oxford, for his continued support throughout this study. We acknowledge the Sir William Dunn School Biosafety Committee for their assistance in risk assessments and advice. This work was supported by a 10.13039/100016302Coalition for Epidemic Preparedness Innovations (CEPI) grant for developing a broadly protective betacoronavirus vaccine.

Schematics in figures were prepared by the help of BioRender and SnapGene. Molecular models shown in Supplementary figure were prepared via ChimeraX.

## Author contributions

M.A, T.K.T., and W.S.J. designed the study. M.H. provided scientific insights on the matters pertaining to risk assessments, biosafety, and biosecurity. M.A. performed all the major experiments and analyzed the data under the supervision of T.K.T. and W.S.J.

D.M. and L.S. prepared recombinant mAbs used in this study under the supervision of T.K.T.

T.C. prepared and provided critical CPER reagents used in this study under the supervision of S.N. and both provided scientific insights for CPER experiments.

M.A. wrote the original draft, and all the co-authors critically reviewed and approved the manuscript.

## Declaration of interests

The authors declare no competing interests.

## STAR★Methods

### Key resources table

**Table:** 

REAGENT or RESOURCE	SOURCE	IDENTIFIER
**Antibodies**		

Recombinant S309 IgG	Pinto et al.^34^	RRID:AB_2941328
Recombinant P4J15 IgG	Fenwick et al.^30^	PDB: 8PQ2
Recombinant S2X259 IgG	Tortorici et al.^33^	PDB: 7RAL
Recombinant C68.59 IgG	Guenthoer et al.^31^	N/A
Recombinant C68.61 IgG	Guenthoer et al.^31^	N/A
Recombinant IY-2A IgG	Huang et al.^29^	PDB: 8HHZ
Recombinant FB-9B IgG	This paper	N/A
Anti-Human IgG (Fc specific)-Peroxidase antibody	Sigma-Aldrich	Cat#A0170; RRID:AB_257868

**Bacterial and virus strains**		

E. coli NEB 5 alpha competent cells	New England Biolabs	Cat#C2987H
SARS-CoV-2 Alpha/Victoria	Gelinas et al.^54^	N/A
SARS-CoV-2 Beta	Gelinas et al.^54^	N/A
SARS-CoV-2 Delta	Gelinas et al.^54^	N/A
SARS-CoV-2 Omicron XBB.1.5	BEI resources	Cat#NR-59104
SARS-CoV-2 Omicron BA.5	African Health Research institute (AHRI)	N/A
SARS-CoV-2 USA-WA1	This paper	N/A
SARS-CoV-2 WA1(JN1 Spike)	This paper	N/A
SARS-CoV-2 WA1(BA.2.86 Spike)	This paper	N/A
SARS-CoV-2 WA1(EG.5.1 Spike)	This paper	N/A
SARS-CoV-2 WA1(XBB.1.16 Spike)	This paper	N/A

**Chemicals, peptides, and recombinant proteins**		

TransIT-LT1 Transfection reagent	Mirus bio	Cat#MIR2300
PrimeSTAR GXL DNA polymerase	Takara Bio	Cat#R050A
HiFi Taq DNA Ligase	New England Biolabs	Cat#M0647S
Q5 site directed mutagenesis kit	New England Biolabs	Cat#E0554S
ExpiCHO Expression kit	Thermofisher Scientific	Cat#A29133
HiTrap MabSelect SuRe	Cytiva	Cat#11003493
Paraformaldehyde 4% in PBS	Thermofisher Scientific	Cat#J61899.AK

**Experimental models: Cell lines**		

HEK293T/17 cells (TMPRSS2+, ACE2+, IFNAR1 KO)	This paper	N/A
Vero E6 TMPRSS2	NIBSC	Cat#100978
Vero (CCL-81)	ATCC	CCL-81
ExpiCHO	Thermofisher Scientific	Cat#A29133

**Oligonucleotides**		

Site directed mutagenesis Forward Primer CTGGTATAGAagcTTTAGGAAGTCTAAACTCAAAC	This paper, Integrated DNA Technologies	N/A
Site directed mutagenesis Forward Primer TAATCATAATTACCACTATGC	This paper, Integrated DNA Technologies	N/A
Primers for CPER, see Table S1	Tori et al.^7^	N/A

**Recombinant DNA**		

PCR-XL-II-TOPO-G1 Plasmid bearing SARS-CoV-2 cDNA fragment	This paper	N/A
PCR-XL-II-TOPO-G2 Plasmid bearing SARS-CoV-2 cDNA fragment	This paper	N/A
PCR-XL-II-TOPO-G3 Plasmid bearing SARS-CoV-2 cDNA fragment	This paper	N/A
PCR-XL-II-TOPO-G4 Plasmid bearing SARS-CoV-2 cDNA fragment	This paper	N/A
PCR-XL-II-TOPO-G5 Plasmid bearing SARS-CoV-2 cDNA fragment	This paper	N/A
PCR-XL-II-TOPO-G6 Plasmid bearing SARS-CoV-2 cDNA fragment	This paper	N/A
PCR-XL-II-TOPO-G7 Plasmid bearing SARS-CoV-2 cDNA fragment	This paper	N/A
PCR-XL-II-TOPO-G8 Plasmid bearing SARS-CoV-2 cDNA fragment	This paper	N/A
PCR-XL-II-TOPO-G9-10 Plasmid bearing SARS-CoV-2 cDNA fragment	This paper	N/A
PCR-Blunt-II-TOPO-GL Plasmid bearing modified Linker fragment	This paper	N/A
F8-XBB.1.16-pMK-RQ Plasmid bearing F8 from SARS-CoV-2 XBB.1.16	This paper	N/A
F8-BA.2.86-pMK-RQ Plasmid bearing F8 from SARS-CoV-2 BA.2.86	This paper	N/A
F8-EG.5.1-pMK-RQ Plasmid bearing F8 from SARS-CoV-2 EG.5.1	This paper	N/A
F8-JN1-pMK-RQ Plasmid bearing F8 from SARS-CoV-2 JN1	This paper	N/A

**Software and algorithms**		

GraphPad Prism	GraphPad software	Prism - GraphPad https://www.graphpad.com/

### Experimental model and study participant details

#### Cell lines and maintenance

Cell lines used in this study include modified HEK293T/17 cells (TMPRSS2+, ACE2+, IFNAR1 KO) cells. These are modified human epithelial kidney (HEK) 293T cells and overexpress human ACE2 and TMPRSS2 and have IFNAR1 knocked out. For routine propagation, these cells were cultured in DMEM with 10% FBS and 1% penicillin/streptomycin in the presence of puromycin (0.001mg/mL final conc.), hygromycin (0.001mg/mL final conc.), and blasticidin (0.01mg/mL final conc.).

For passaging, cells were first washed with phosphate buffered saline, and then detached with TryplE Express.

Vero E6 cells were cultured in DMEM with 10% FBS and 1% penicillin/streptomycin.

VeroE6 TMPRSS2 cells were cultured in 10% FBS and 1% penicillin/streptomycin and Genticin (G418) 1mg/ml. All of the above adherent cells were cultured at 37°C in 5% CO_2_.

ExpiCHO cells were cultured in EXPI medium as suspension cultures while shaking at 120 rpm at 37°C in 8% CO_2_.

### Method details

#### Risk assessments for biosafety and biosecurity

The CPER reverse genetics work was subject to UK Health and Safety Executive consent and University of Oxford risk assessment approval. All research work with SARS-CoV-2 WT and CPER viruses was carried out in Containment Level 3 (CL3) facility where biohazard control measures are in place including safe working practices to prevent uncontrolled release of these viruses. Considering the dual use risks associated with such research,^55^ we have only briefly described the modifications to the original protocol and referred to the previously published methods wherever possible.

#### Generation of SARS-CoV-2 virus by CPER reverse genetics

The original CPER method has been described in detail.^7^ An overview of all the steps in the protocol are shown in Figure 1. Key steps of the method are described below.

#### Plasmids

SARS-CoV-2 genome cDNA overlapping fragments were based on the Torii et al’s genome segmentation scheme. Plasmids bearing spike gene fragment F8 for SARS-CoV-2 omicron variants XBB.1.16, EG.5.1, BA.2.86 were commercially synthesized. Plasmid bearing spike gene fragment F8 for SARS-CoV-2 Omicron variants JN.1 was prepared by site directed mutagenesis.

*E. coli* NEB 5 alpha competent cells were transformed with these plasmids and the transformed cells were cultured in LB broth supplemented with the antibiotics (Kanamycin for plasmid bearing Linker fragment as well as the plasmids bearing F8 i.e. spike gene of recent variants. Ampicillin for plasmids bearing F1-F7 and F9 of SARS-CoV-2) for miniprep.

#### Circular polymerase extension reaction

SARS-CoV-2 USA-WA1 genome segmentation scheme based on nine overlapping fragments as shown in Figure 3A. The plasmids bearing cDNA, corresponding to the SARS-CoV-2 genome fragments, as well as a modified linker fragment bearing 3′UTR of SARS-CoV-2, antigenomic ribozyme HDV 1, SV40 3′ Splice site, SV40 poly(A) 1, SV40 ori, and CMV promoter, and 5′end of SARS-CoV-2 genome were used to prepare the CPER fragments. PCR was done to amplify each DNA fragment using the PrimeSTAR GXL DNA polymerase (Takara Bio), the appropriate primer pairs^7^ and plasmid template DNA. Both 5′ phosphorylated primers as well as unphosphorylated primers were used in separate reactions. PCR conditions for 3-step PCR using rapid protocol were: 35 cycles of 98°C for 10 sec, 60°C for 15 sec, 68°C for 1 min 30 sec. PCR product bands were resolved and visualised on agarose gel which were then extracted with QIAquick gel extraction kit and quantified on a nanodrop. CPER reaction was set up using equimolar quantities of each fragment and was run using the following program 98°C for 2min (Initial denaturation); 35 cycles of 98°C for 10sec, 55°C for 15sec and 68°C for 15min followed by 68°C for 15min (final extension).

#### Nick ligation

The CPER reactions from 5′ phosphorylated primers (Figure 1B) were treated with thermostable DNA ligase for 30 min at 50°C and 30 min at 60°C in the 50uL reaction containing 1uL HIFI Taq DNA ligase and NAD+. In the initial experiment, the CPER reaction using unphosphorylated primers (Figure 1C) were not ligated and represent the unsealed/classical CPER.

#### Transfection & co-culture

HEK293T/17 (ACE2+, TMPRSS2+, IFNAR1 KO) cells were transfected with the nick ligated or unsealed CPER reactions in 6 well plates using TransIT-LT1 transfection reagent and OptiMEM serum free medium. The medium in the 6 well plates was replaced with 2% FCS DMEM 24 hours after transfection. Transfected cells were then co-cultured with VeroE6 TMPRSS2 cells in T25 flasks on 3^rd^ day post transfection. The flasks were monitored for cytopathic effects (CPE) every day for the next 5 days, and samples were collected when the CPE were visible. Samples of the culture supernatant collected on 4^th^, 5^th^, and 6^th^ day post-co-culture were titrated with the Focus forming unit (FFU) assay based on immunostaining of SARS-CoV-2 N protein to determine the virus titre (Figure 1D).

In a second set of experiments, we used phosphorylated primers to generate the genome fragments used in both the nick ligated and the unsealed CPER reactions to see if cellular ligases can seal the nicks readily if the DNA is provided with 5′phosphate groups (Figure 2).

We have also scaled up the co-culture experiments in T75 flasks to optimise the titre of the rescued virus (Figure S4B). For comparison, we have also performed a direct transfection of VeroE6 TMPRSS2 cell line with the nick ligated CPER product as described previously.^10^ A direct comparison is not possible as we have used our modified linker fragment in CPER assembly (Figure S4).

#### Generation of spike-chimeric viruses

Public databases NCBI Virus, GISAID, NEXT STRAIN etc were used to acquire the SARS-CoV-2 emerging variant genomic data. Complete sequence was retrieved from NCBI for Omicron BA 2.86 (GenBank: OR558991.1), Omicron JN.1 (GenBank: OR976795.1), Omicron EG 5.1 (GenBank: BS007377.1) and Omicron XBB.1.16 (GenBank: BS007330.1). Sequence analysis of emerging variants was done for identification of spike protein mutations in existing epitopes targeted by neutralising antibodies, Protein sequence analysis revealed the amino acid variations that were to be incorporated in spike gene (based in Fragment 8 of CPER assembly). Either the spike gene was synthesised commercially (for Omicron XBB.1.16, BA.2.86 and EG.5.1) or else site directed mutagenesis was used to create RBD mutations (for Omicron JN.1). Site directed mutagenesis for JN.1 was done using the BA.2.86 spike plasmid as Template DNA and the mutagenic primers (Forward Primer CTGGTATAGAagcTTTAGGAAGTCTAAACTCAAAC and Reverse Primer TAATCATAATTACCACTATGC) to introduce the L455S mutation using Q5 site-directed mutagenesis kit. This was followed by nanopore sequencing for sequence confirmation. (See Figure S1) The F8 for JN.1 was then PCR amplified using the mutation confirmed plasmid. The CPER assembly in all the cases was performed using the F8 of emerging variant with other fragments from the USA-WA1 backbone, as described in Figure 3A. The CPER RG viruses were harvested when the VeroE6 TMPRSS2 cells showed cytopathic effects and were titrated with the FFU assay as shown in Figure 3.

These RG engineered viruses were also passaged on VeroE6 TMPRSS2 cells to ensure the infectivity.

#### Focus forming unit assay

FFU assays based on CMC-overlay method we performed as described previously.^56^ Serial dilutions of the harvested virus were prepared with DMEM medium supplemented with 1% FBS. Then 20 uL of each dilution was added in triplicate in 3 wells of a 96 well cell culture plate. Then 100 μL Vero CCL81 cells (4.5 × 10^4^) were added and incubated at 37°C, 5% CO_2_. After 2 hours, 100 μL of a 1.5% carboxymethyl cellulose-containing overlay was applied to prevent satellite focus formation. The plates were incubated until 20 hours post-infection and then the overlay was aspirated, and wells were gently washed with 180 μL PBS to remove traces of serum proteins and amino acids that might interfere with aldehyde fixation. Then the monolayers were fixed with 4% paraformaldehyde at room temperature for 30 minutes, and were permeabilised with 100 μL permeabilization buffer (2% Triton-X 100 in PBS) at 37°C for 30 minutes. The plates were rinsed 3 times with 100 μl of washing buffer (0.1 % Tween 20 in PBS) and stained for SARS-CoV-2 Nucleocapsid protein with anti-N FB9B primary antibody (Sigma-Aldrich Cat# A0170, RRID:AB_257868) diluted 1:2000 in the washing buffer for 1 hour at room temperature while shaking. The plates were rinsed 6 times with washing buffer. Anti-human IgG-HRP antibody was diluted 1:5,000 in the washing buffer and added to every well. The plates were developed with TrueBlue peroxidase substrate at room temperature for 10 minutes. The reaction was stopped with distilled water, and the plates were dried. The foci were counted on an ELISPOT reader. Virus titres were calculated as FFU/mL =Average of the FFU count in a readable triplicate x 50 x dilution factor.

#### Recombinant expression of mAbs

Recombinant mAbs were synthesized using the publicly available VH & VL sequences. The codon optimised genes encoding the heavy and light chains of the monoclonal antibody were cloned into the human IgG1 heavy chain and kappa or lambda light chain expression plasmids under the control of CMV promoters. ExpiCHO cells were transiently transfected with the plasmids using ExpiFectamine transfection reagent and were incubated at 37°C with 8% CO_2_ and 120 rpm. One day post-transfection, the cultures were treated with the enhancer according to the manufacturer’s instructions and incubated at 32°C with 5% CO_2_. After 5-7 days the cells were harvested by centrifugation, and the supernatant was collected for downstream purification. The mAbs were purified from the harvested supernatant via affinity chromatography using an HiTrap MabSelect SuRe antibody purification column according to the manufacturer’s instructions. Purified antibodies were analysed on SDS-PAGE to confirm the purity.

#### Virus neutralisation assays

Virus neutralisation assays based on CMC-overlay method were performed as described previously.^54^^,^^56^^,^^57^ Alpha (B.1.1.7), Beta (B.1.351), Delta (B.1.617.2), and Omicron (B.1.1.529) subvariants BA.5, XBB.1.5, XBB.1.16, EG.5.1, BA.2.86 and JN.1 were selected for analysis of neutralisation potency of the mAbs. Alpha (B.1.1.7 obtained from Public Health England, Porton Down),^54^ Beta (B.1.351 obtained from the Center for the AIDS Program of Research in South Africa),^54^ Delta (B.1.617.2 obtained from Laboratory of Clinical and Epidemiological Virology, Rega Institute, KU Leuven),^54^ and Omicron (B.1.1.529) subvariants BA.5 (obtained from African Health Research Institute) and XBB.1.5 (obtained from BEI resources) were all natural isolates whereas Omicron subvariants XBB.1.16, EG.5.1, BA.2.86 and JN.1 were reverse genetic engineered spike chimeric viruses as described above. Briefly, serial dilutions of mAbs were mixed with the SARS-CoV-2 variants in 96 well plates and incubated at room temperature for 30 minutes. After 30 minutes, Vero CCL81 cells were added to the plates, and the plates were incubated at 37°C for 2 hours. Then the plates were overlaid with 1.5% CMC overlay and the plates were incubated at 37°C for 22 hours. The overlay was carefully removed and the plates were fixed with 4% paraformaldehyde. The cells in fixed plates were then immunostained with HRP labelled FB9B antibody (Sigma-Aldrich Cat# A0170, RRID:AB_257868) which binds to N protein of SARS-CoV-2.

### Quantification and statistical analysis

The microneutralisation assay was performed in triplicates. Plates were analysed on AID ELISpot Reader System for counting the foci. Data were analysed using four-parameter logistic regression (Hill equation) and the IC50 values were calculated using GraphPad Prism (GraphPad Software V.10.2).

## Supplemental information

Document S1. Figures S1–S4

Table S1. Primers used for amplification of SARS-CoV-2 CPER fragments as described by Torii et al., related to STAR Methods We modified these primers with a 5′phosphate group to facilitate nick ligation. Uppercase letters indicate the sequence homologous to the Linker DNA and lowercase sequence anneals to the cDNA of SARS-CoV-2 genome.
