# Supplementary material for: Determination of resilience of a panel of broadly neutralizing mAbs to emerging variants of SARS-CoV-2 generated using reverse genetics
Source: iScience. 2025 Apr 16;28(6):112451. doi: 10.1016/j.isci.2025.112451 (PMC12150062; doi:10.1016/j.isci.2025.112451)
Supplement: Document S1. Figures S1–S4 [file mmc1.pdf]

iScience, Volume *28*

Supplemental information

Determination of resilience of a panel of broadly neutralizing mAbs to emerging variants of SARS-CoV-2 generated using reverse genetics

Madeeha Afzal, Diana Melnyk, Thomas Courty, Lisa Schimanski, Michelle Hill, Stuart Neil, Tiong Kit Tan, and William S. James

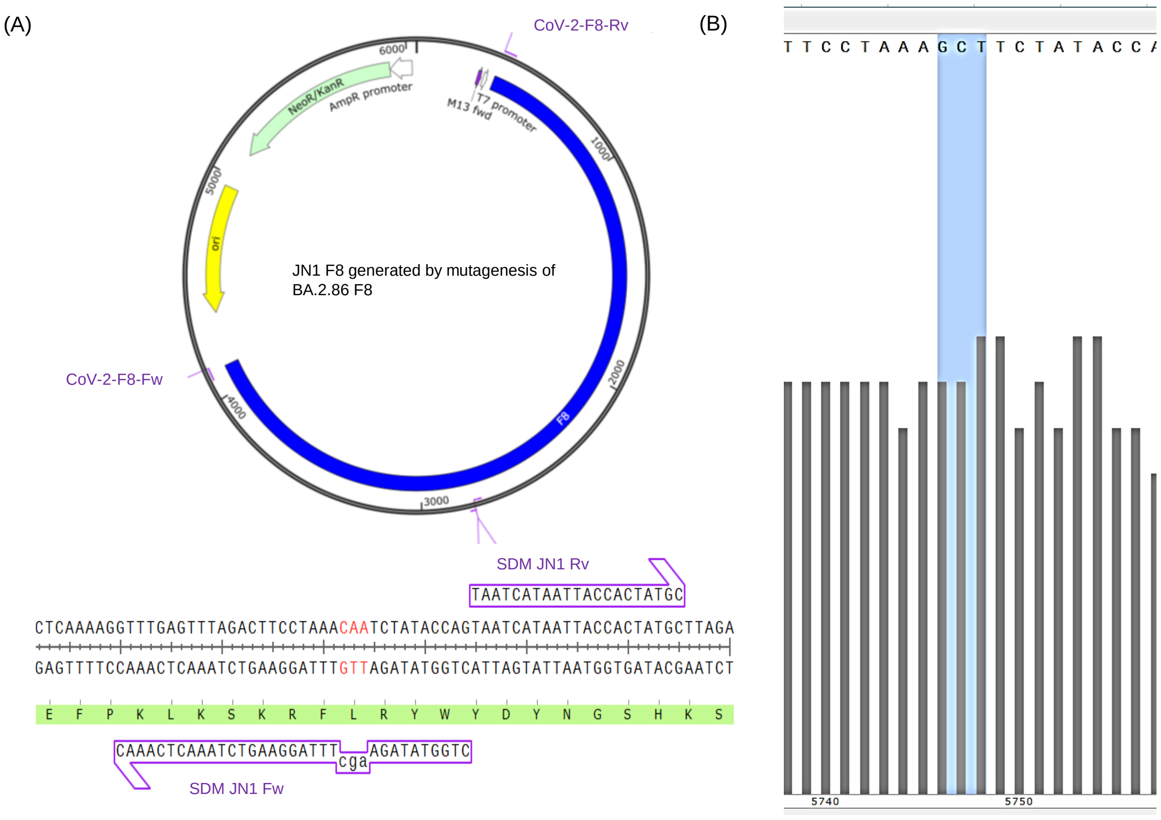

Figure S1. Site directed mutagenesis of BA2.86 Spike fragment for generating L455S mutation. (A) Mutagenic primers binding sites on F8-BA.2.86-pMK-RQ plasmid bearing F8 from SARS-CoV-2 BA.2.86 (B) Nanopore sequencing confirms mutagenesis, related to Figure 3.

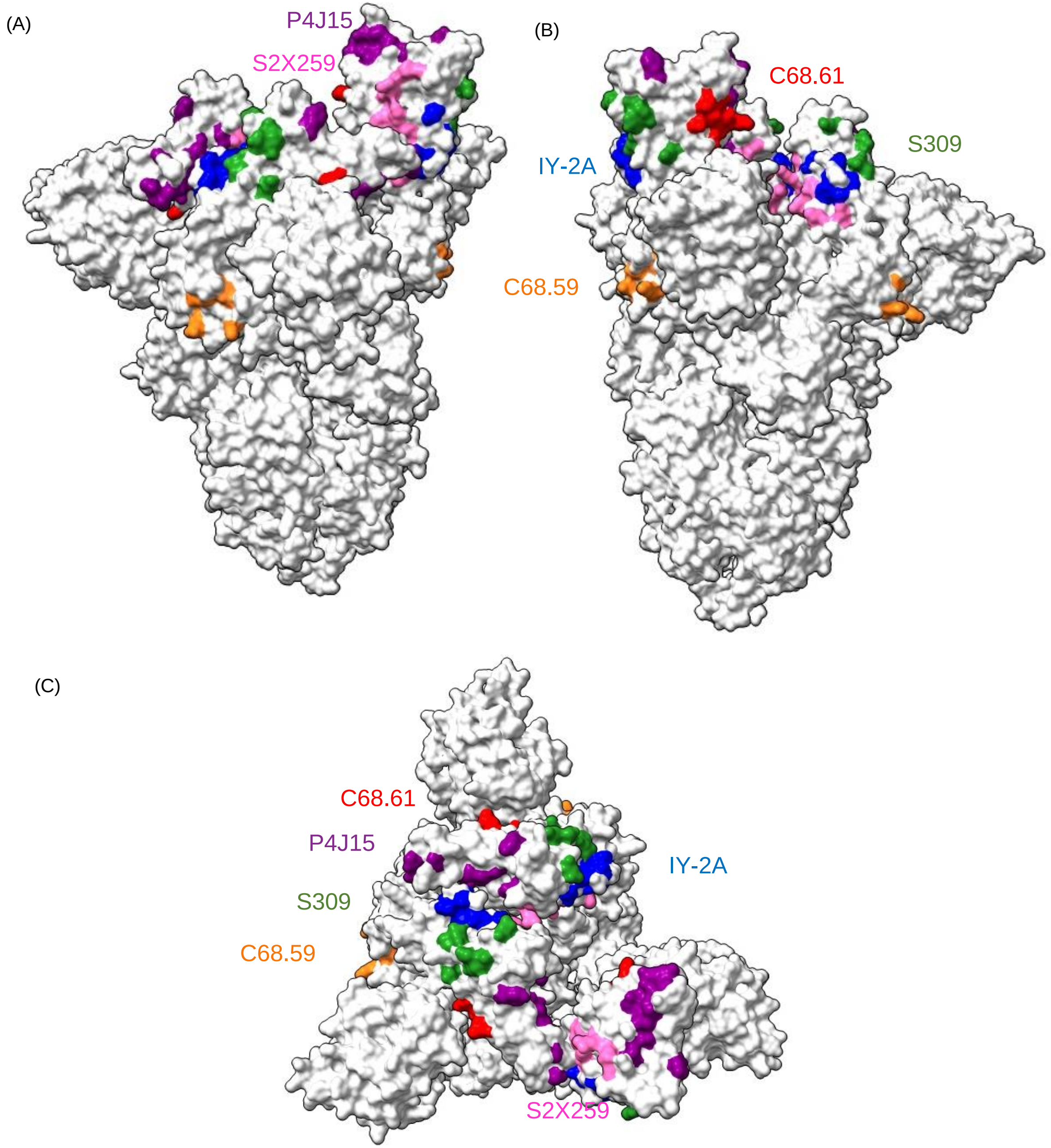

Figure S2. Molecular models of spike trimer in one-up and two-down conformation. Epitope of C68.61 in RBD (red), IY-2A in RBD (blue), C68.59 in SD1 (orange), S309 in RBD (green), S2X259 in RBD (pink) and P4J15 in RBD (purple). (A) front view (B) back view and (C) top view, related to Figure 5.

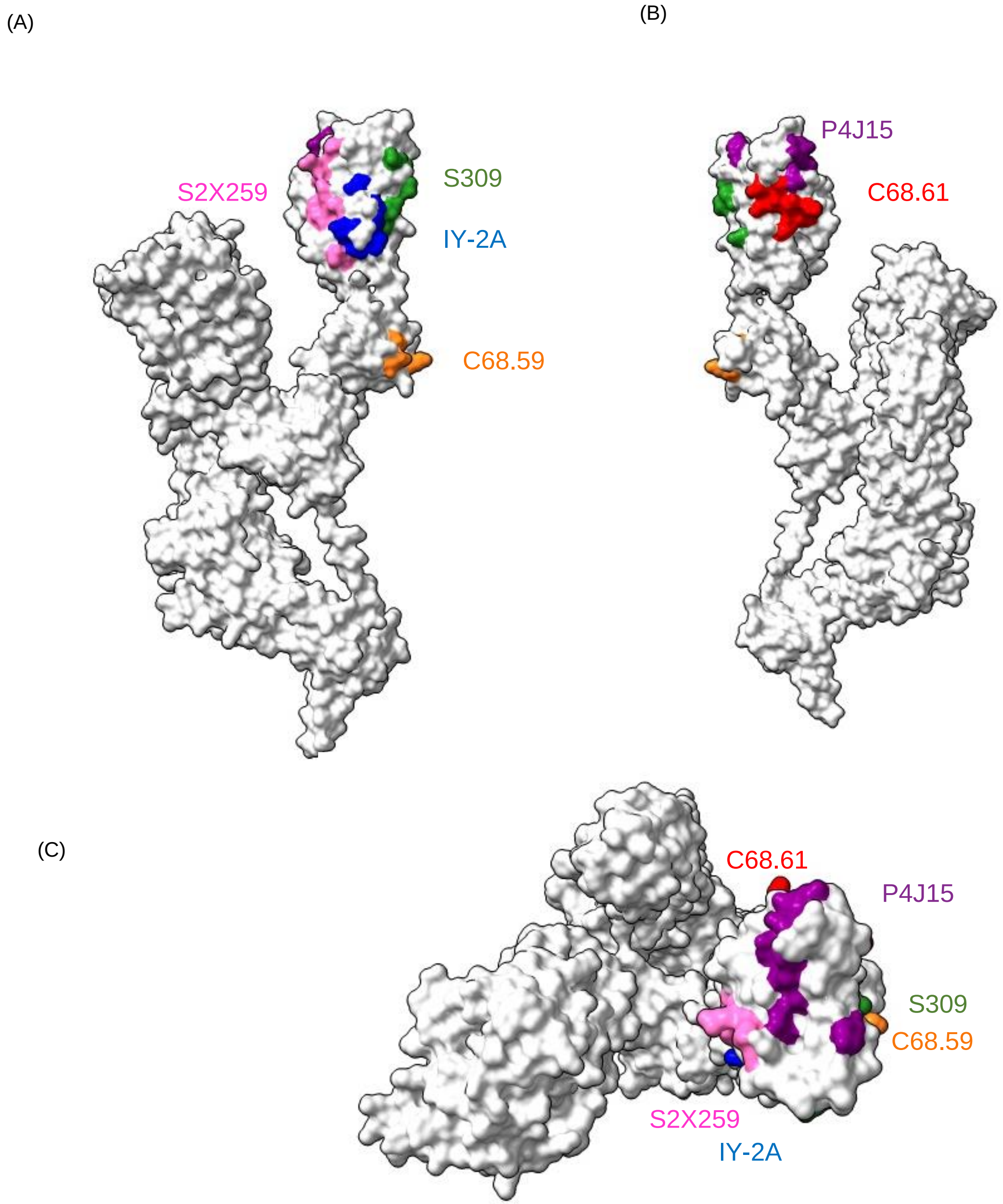

Figure S3. Molecular models of spike monomer indicating binding sites of mAbs. Epitope of C68.61 in RBD (red), IY-2A in RBD (blue), C68.59 in SD1 (orange), S309 in RBD (green), S2X-259 in RBD (pink) and P4J15 in RBD (purple). (A) front view, (B) back view and (C) top view, related to Figure 5.

(A) Vero E6 TMPRSS2 cells in 6 well plates, transfected with USA-WA1 Nick-ligated CPER (top panel) and mock transfected (bottom panel).

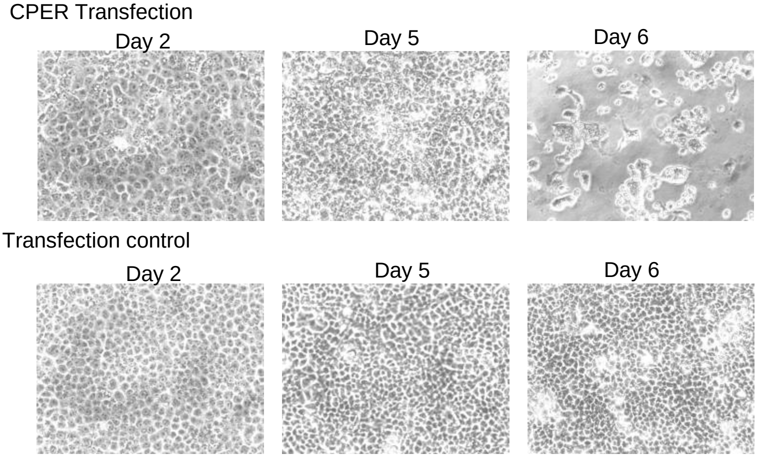

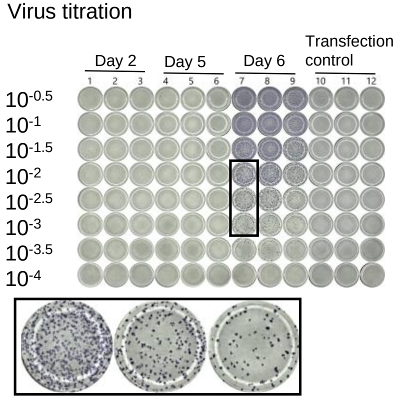

VIRUS SAMPLE	TITRE (FFU/ml)
Nick ligated CPER, day 6 post transfection	$1x10^6$

(B) Optimisation of CPER co-culture for large scale virus rescue. Modified HEK TAI cells transfected with nick-ligated CPER, and co-cultured with Vero E6 TMPRSS2 cells in T75 flasks.

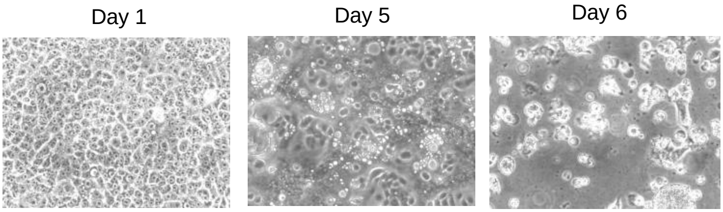

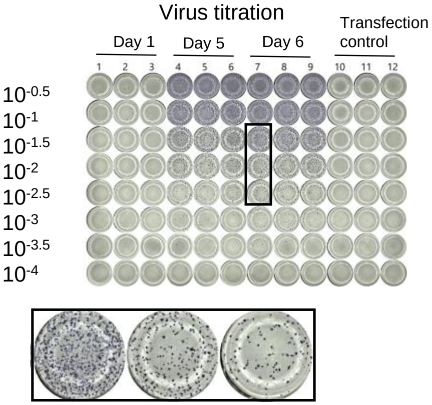

VIRUS SAMPLE	TITRE (FFU/ml)
Nick ligated CPER, day 5 post co-culture	$8x10^5$
Nick ligated CPER, day 6 post co-culture	$1x10^6$

Figure S4. Comparison of direct transfection of VeroE6 TMPRSS2 cells versus the co-culture of VeroE6 TMPRSS2 cells with the transfected HEK293T17 (A) Direct transfection of Vero E6 TMPRSS2 cells with CPER products was able to generate virus on day 6 post transfection (B) CPER co-culture for large scale virus rescue was able to generate high titre virus on day 5 post co-culture, related to Figure 2.
