# Supplementary material for: Determination of resilience of a panel of broadly neutralizing mAbs to emerging variants of SARS-CoV-2 generated using reverse genetics
Source: iScience. 2025 Apr 16;28(6):112451. doi: 10.1016/j.isci.2025.112451 (PMC12150062; doi:10.1016/j.isci.2025.112451)
Supplement: Table S1. Primers used for amplification of SARS-CoV-2 CPER fragments as described by Torii et al., related to STAR Methods — We modified these primers with a 5′phosphate group to facilitate nick ligation. Uppercase letters indicate the sequence homologous to the Linker DNA and lowercase sequence anneals to the cDNA of SARS-CoV-2 genome. [file mmc2.pdf]

SUPPLEMENTARY TABLE S1 Primers used for amplification of SARS-CoV-2 CPER fragments as described by Torii et al. We modified these primers with a 5'phosphate group to facilitate nick ligation. Uppercase letters indicate the sequence homologous to the Linker DNA and lowercase sequence anneals to the cDNA of SARS-CoV-2 genome.

Primer Name	Sequence
CoV-2-F1-Fw	CTATATAAGCAGAGCTCGTTTAGTGAACCGTattaaaggtttataccttcccaggtaac
CoV-2-F1-Rv	cagattcaacttgcatggcattgttagtagccttatttaaggctcctgc
CoV-2-F2-Fw	gcaggagccttaaataaggctactaacaatgccatgcaagttgaatctg
CoV-2-F2-Rv	ggtaggattttccactacttcttcagagactggttttagatcttcgcaggc
CoV-2-F3-Fw	gcctgcgaagatctaaaaccagtctctgaagaagtagtggaaaatcctacc
CoV-2-F3-Rv	ggtgcacagcgcagcttcttcaaaagtactaaagg
CoV-2-F4-Fw	caccactaattcaacctattggtgctttggacatatcagcatctatagtagctggtgg
CoV-2-F4-Rv	gtttaaaaacgattgtgcatcagctgactg
CoV-2-F5-Fw	cacagtctgtaccgtctgcggtatgtggaaaggttatggctgtagttgtgatc
CoV-2-F5-Rv	gcggtgtgtacatagcctcataaaactcaggttcccaataccttgaagtg
CoV-2-F6-Fw	cacttcaaggtattgggaacctgagttttatgaggctatgtacacaccgc
CoV-2-F6-Rv	catacaaactgccaccatcacaaccaggcaagttaaggttagatagcactctag
CoV-2-F7-Fw	ctagagtgctatctaaccttaacttgcctggttgtgatggtggcagtttgtatg
CoV-2-F7-Rv	ctagagactagtggcaataaaacaagaaaaacaaacattgttcgtttagttgttaac
CoV-2-F8-Fw	gttaacaactaaacgaacaatgtttgtttttcttgttttattgccactagtctctag
CoV-2-F8-Rv	gcagcaggatccacaagaacaacagcccttgagacaactacagcaactgg
CoV-2-F9-Fw	ccagttgctgtagttgtctcaagggctgttgttcttgtggatcctgctgc
CoV-2-F10-Rv	GGAGATGCCATGCCGACCCtttttttttttttttttttttttttgtcattctcctaag
CoV-2-Linker-Fw	cttaggagaatgacaaaaaaaaaaaaaaaaaaaaaaaaaGGGTCGGCATGGCATCTCC
CoV-2-Linker-Rv	gttacctgggaaggtataaacctttaatACGGTTCACTAAACGAGCTCTGCTTATATAG

SUPPLEMENTARY REFERENCES

(1) Torii, S., Ono, C., Suzuki, R., Morioka, Y., Anzai, I., Fauzyah, Y., Maeda, Y., Kamitani, W., Fukuhara, T., and Matsuura, Y. (2021). Establishment of a reverse genetics system for SARS-CoV-2 using circular polymerase extension reaction. Cell Rep *35*, 109014. 10.1016/j.celrep.2021.109014.
